# Functional absence of flexor digitorum superficialis of the 5^th^ digit and its association with agenesis of palmaris longus

**DOI:** 10.12669/pjms.41.7.10559

**Published:** 2025-07

**Authors:** Quratulain Javaid, Loung Umedani

**Affiliations:** 1 Quratulain Javaid, MBBS, PGD-Bioethics, MPhil-Anatomy Department of Anatomy, Bahria University Health Sciences, Campus Karachi, Pakistan; 2 Loung Umedani, MBBS, PhD. Department of Biochemistry, Bahria University Health Sciences, Campus Karachi, Pakistan

**Keywords:** Anatomical agenesis, Functional, absence, Flexor digitorum superficialis, Palmaris longus

## Abstract

**Objective:**

To determine the prevalence of absence of Flexor digitorum superficialis and its association with gender, handedness, and agenesis of Palmaris Longus

**Methods:**

A cross-sectional descriptive study was carried out at Bahria University Health Sciences Campus (BUHSC), Karachi from August 2022 to September 2024 that included both males and females. Standard and Modified Tests for Flexor digitorum superficialis (FDS) while Schaffer’s and Thompson’s Test for Palmaris longus PL were used to check prevalence. By SPSS version 27, statistical analysis was done. Frequencies and percentages were used to show categorical variables. To determine the association of the absence of FDS superficialis with gender, handedness, and agenesis of PL, the Chi-square test was used. A P-value of ≤0.05 was considered as significant.

**Results:**

Five hundred and four limbs were observed for functional agenesis of FDS for the 5^th^ digit and agenesis of PL. Overall, functional agenesis of FDS for the 5^th^ digit was 35.7% while PL agenesis was 33.7%. Functionality of FDS showed highly significant results (p<0.001) depicting variability on two sides in terms of dependent and independent function. When handedness was compared, results were not statistically significant for FDS absence (p=0.73). When agenesis of FDS was compared with PL, results were not statistically significant (p=0.527). Bilateral agenesis of both PL and FDS was 3.2% while unilateral agenesis of both muscles was 10%.

**Conclusion:**

FDS functionality showed variability on two sides in terms of dependent and independent function. Both genders showed a higher prevalence of right-sided agenesis. Females showed more unilateral agenesis while males had higher bilateral prevalence. We found no association between agenesis of the FDS of 5^th^ digit with handedness and PL agenesis.

## INTRODUCTION

The flexor digitorum superficialis (FDS) and palmaris longus (PL) are members of the superficial group of forearm flexors. The common origin of both muscles is from the medial epicondyle of the humerus.^1^ Additionally, FDS also arise from oblique line of radius and ulna’s coronoid process. FDS consists of four tendons that pass on the deeper aspect of flexor retinaculum, and then later the attachment is on the shaft of middle phalange of four fingers. Functionally, mainly it flexes the proximal interphalangeal joints but it also works as a flexor for joints proximal to its insertion. FDS variations include absence of its tendon for 5th finger (most common anomaly), variability of tendon number, mode of its tendon insertions and tendinous interconnections. In the adults, PL has a short belly and a long tendon inserted into the flexor retinaculum followed by merging with the palmar aponeurosis.^1^-^3^ From a phylogenetic aspect it is a degenerating muscle while functionally it is a weak flexor of wrist and digits.^4^

The absence of 5^th^ digit FDS function varies widely geographically ranging from 0% in the Indian population to 30.77% in the British population.^5^ The variability exists also when the male and female genders are compared in terms of laterality, handedness, and ethnicity.^6^ Also, hypoplasticity of the FDS tendon was also reported.^7^ An absent FDS tendon affects the performance of string player musicians. A combined absence of 4^th^ and 5^th^ digit FDS tendons markedly affects fine motor activity like playing music.^8^ Anatomically, the organization of FDS tendons contributes to preference for one’s left-handedness. In a study carried out on 236 hands from 118 subjects, the left-hand dominance was observed in 5% of individuals. In these cases, the absence of the fifth finger FDS tendon in the right hand was higher as compared to right-hand subjects (71.4% vs. 28.8%) respectively.^9^ In a study carried out on 619 subjects from the Urban area of Punjab province of Pakistan, the PL and FDS absence was found as 49.3% and 23.6% respectively.^10^

Considering that both FDS and PL have a similar origin, nerve supply, and common function, it could be hypothesized that their agenesis could be similar or associated to each other. Research done across the globe has mentioned variability in terms of the association between agenesis of FDS and PL. Shah et al have mentioned the absence of PL along with the underdevelopment of FDS^11^ while some researchers have mentioned no statistically significant association of absence between the two muscles.^2^,^10^,^12^ The superficial location of FDS and PL make them a choice for plastic surgeries and other reconstructive and graft procedures.^13^ Both FDS and PL can be considered among the muscles that exhibit variable anatomy and hence, the unique and unusual pattern of both muscles must be known to the physiotherapists, surgeons, and anatomists.

The variability in anatomy can have effects on the results of physical examination and differential diagnosis.^14^ Hence, it is mandatory to know about the distinctive pattern of both muscles and confirm the physical existence by performing clinical tests. As the research about PL and FDS physical and functional parameters indicated above in Pakistan was scanty and as it mainly addressed some particular parts of the country, we planned to conduct the present study in the biggest city of Pakistan that harbors multiple ethnicities. The main focus was to determine the prevalence of the absence of flexor digitorum superficialis and its association with male and female gender, handedness, and agenesis of Palmaris Longus.

## METHODS

A cross-sectional descriptive study was carried out at Bahria University Health Sciences Campus (BUHSC), Karachi from August 2022 to September 2024. The participants were recruited by non-randomized convenience sampling.

### Ethical approval:

Ethical approval was taken from Ethical Review Committee (ERC) of BUHSCK, reference number (ERC-14/2022, dated May 16, 2022). Informed consent was taken from the participants before recruitment in the study and those who willingly participated were made part of the study.

A sample of 252 that included both males and females was drawn from students of BUHSC. The observed age range of the study participants was from 18-23 years. OpenEpi version 3 calculator was used to calculate the sample size based on population prevalence of 50%, margin of error 5% and confidence interval (CI) of 95% with an estimated agenesis of 20.6%.^5^

### Inclusion criteria & Exclusion criteria:

Participants were eligible if they had no prior history of injuries, fractures, or surgical interventions involving the forearm or hand. Subjects were excluded if they had a documented history of trauma, congenital or acquired deformities, or surgical procedures affecting the forearm or hand.

Standard Test and Modified Test were used to observe the prevalence of absence of FDS.^10^ While doing the Standard Test, participants were asked to extend all fingers (except for the digiti minimi) fully which involved the extension of both proximal interphalangeal and distal interphalangeal joints. FDS was considered positive if there was flexion at the proximal interphalangeal joint of digiti minimi. A modified test was used to check whether there exists any interconnection of tendons between the ring and the little finger. It was considered positive when there was flexion at the proximal interphalangeal joint of digiti minimi on extension of both proximal interphalangeal joints and distal interphalangeal joints of index and middle finger. Palmaris longus was tested by two tests; Schaffer’s Test^15^ and Thompson’s test^16^. Schaffer’s Test involved bringing the palmar aspect of thumb and little finger together and flexing the wrist joint. The palmaris longus tendon if present becomes visible.^2^ In case it was absent, Thompson’s test was performed to verify; it involved making a fist and flexing the wrist joint.^3^

### Statistical analysis:

SPSS version 27, was used for statistical analysis was done. To check normality of data, Kolmogorov–Smirnov test was used. Frequencies and percentages were used to show categorical variables. To determine association of prevalence of absence of flexor digitorum superficialis with male and female gender, handedness and agenesis of Palmaris Longus, Chi-square test was used. P-value of ≤0.05 was considered as significant.

## RESULTS

**Fig.1:**
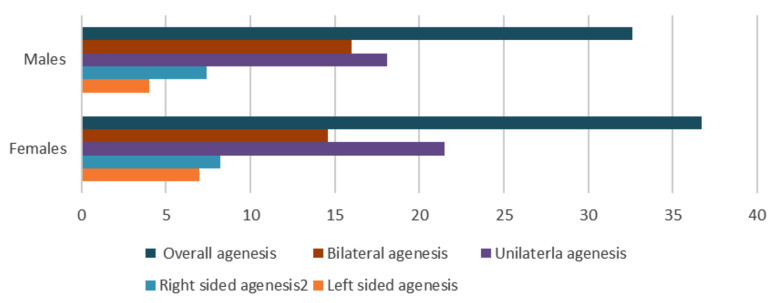
Prevalence of FDS agenesis between the two genders.

A total of 504 limbs were observed for functional agenesis of FDS for the 5^th^ digit and agenesis of PL. Mean age of the research participants was 20.03±1.08 years. The study included 252 participants, comprising 156 females (61.9%) and 96 males (38.1%). Functional agenesis of the 5th digit was observed in 90 out of 252 individuals (35.7%), while palmaris longus agenesis was identified in 85 out of 252 participants (33.7%). Overall, functional presence was noted in 166 out of 252 participants (65.9%), with 66 out of 96 males (67.4%) and 100 out of 156 females (63.3%) exhibiting functional presence. A significant difference in agenesis rates was observed between genders (Fig. 1). Functionality of FDS showed highly significant results (p<0.001) (Table-I) depicting variability on the two sides in terms of dependent and independent function.

**Table-I:** Functionality of FDS according to side of the limb.

	Side of Forearm		P value
Type of function	Right	Left	
Independent	120 (47.6%)	122 (48.4%)	p<0.001
Dependent	74(29.3%)	81 (32.1%)	
Absent	58 (23%)	49 (19.4%)	

When handedness was compared, the results were not statistically significant for FDS absence (p=0.73) (Fig.1). Our study showed that 241 (95.6%) were right handed while 11 (4.3%) were left handed. When bilateral agenesis was compared, it was 36 (14.9%) and 3 (27.3%) in right and left handed participants respectively. Unilateral agenesis was somewhat similar in the right 49 (20.3%) and left 2 (18.2%) sided study subjects. Right sided agenesis was observed in 20 (8.3%) right handed subjects while none from left handed showed this type of agenesis. In terms of left sided agenesis, it was observed in 14 (5.8%) right handed and 1 (9.1%) in the left handed individuals.

**Fig.2:**
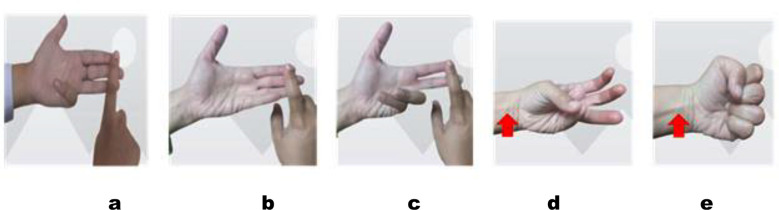
Clinical Tests to check FDS and PL prevalence. a: Standard test with positive FDS, b: Standard test with FDS absence, c: Modified test with FDS presence, d: Schaeffer’s test with PL presence e: Thompson’s test with PL presence

When agenesis of FDS was compared with PL, the results were not statistically significant (p=0.527) (Table-II). In 08 (3.1%) there was bilateral agenesis of both PL and FDS while unilateral agenesis of both muscles was in 25 (10%). Right and left agenesis was in 12 (4.7%) and 13 (5.1%) respectively.

**Table-II:** Comparison of FDS and Palmaris Longus Agenesis.

	Palmaris Longus			P value
FDS	Unilateral agenesis	Bilateral agenesis	Both Present	
Unilateral agenesis	17 (33.3%)	08 (15.7%)	26 (51%)	0.527
Bilateral agenesis	09 (23.1%)	07 (17.9%)	23 (59%)	
Both present	46 (28.4%)	39 (24.1%)	77 (47.5%)	

## DISCUSSION

Anatomical variations exist in terms of two forearm muscles; palmaris longus and flexor digitorum superficialis.^17^,^18^ Our study showed overall functional agenesis of 5^th^ finger’s FDS to be 35.7%. Vucinic et al has mentioned similar agenesis prevalence (33.3%).^12^ Rafique et al has stated the total agenesis to be 26.1% using combined absence checked by both the Standard and Modified tests.^10^ A study conducted in India has showed lesser absence prevalence and mentioned the agenesis to be 18%.^2^ Comparatively lesser prevalence (15% and 14.8%) was documented in Bulgaria and Saudi Arabia based studies respectively.^3^,^13^ No agenesis was reported in a study conducted in Karnataka, India.^18^ These disparities underscore the influence of geographical and ethnic factors on the prevalence of FDS agenesis.

The current study has showed no statistically associated differences between gender and FDS agenesis. Our study has mentioned more bilateral agenesis in males whereas in the females more unilateral agenesis was observed. The observations were in line with the meta-analysis documented by Yammine and Eric.^6^ The pattern of FDS could be due to embryological differences in the development between the two genders. The current study suggests that right sided agenesis is more than left-sided agenesis in both male and female genders. Guler et al has mentioned parallel results.^19^ The right sided hand dominance could be a contributing factor towards high reporting of right sided agenesis. Similar to our findings, a study conducted in Turkey has stated no statistically significant association between males and females in terms of FDS agenesis. Also, they have mentioned more FDS agenesis for females (24.6%) as compared to males (22.7%). When laterality was compared, statistically significant association was stated for bilateral agenesis and unilateral agenesis.^20^ A study conducted in Bulgaria also witnessed more female prevalence (22%) than males (8%).^3^ Alzahrani et al has documented more female FDs agenesis (14.3%) on the left sides for females as compared to males (4.8%). On the right limb, males (12.8%) reported more than females (11.3%).^13^ Comparable results in terms of female high occurrence but with high prevalence percentage were stated by a Brazil based study. On the right side, females were reported to have 37.10% against males (23.72%) while on the left side the percentages were 40.26% and 29.38% for females and males respectively.^5^

Our study showed more independent function as compared to dependent function of FDS and the results were statistically significant when the two sides were compared. Similar to our results, other studies have also stated lesser prevalence of dependent FDS tendon between the 4^th^ and 5^th^ digits. A study conducted in Poland has stated more prevalence of independent function (62.5%) and dependent function to be comparatively lesser (29.9%).^21^ A study conducted in India has also showed similar results and stated the dependent function to be 64% and 66.5% respectively on the right limb as compared to 16.5% and 17% respectively for dependent function on the left limb.^2^ Rafique et al have mentioned 75.4% prevalence for independent tendon of FDS while 22.5% of participants have dependent functions.^10^ A study conducted in Japan has mentioned that independent function was in 67.5% as compared to 32.5% dependent function.^22^ Yammine et al have documented 37.5% study subjects to have shared tendons functionality.^6^

The present study showed statistically non-significant relation between FDS absence and handedness. Among the left-handers, bilateral and left sided agenesis was more prevalent while in right-handed individuals right sided agenesis was prevalent. Similar to our results, Heshmati et al have documented no correlation between agenesis of FDS and handedness.^23^ However, Alzahrani et al. found a higher prevalence of FDS agenesis among right-handed study participants (88%) and documented a statistically significant association between FDS absence and handedness.^13^ The incongruities between studies may be attributed to differences in sample sizes, population demographics, or assessment methodologies.

The present study observed no statistically significant association between the agenesis of FDS and PL tendons. This corresponds to the findings from a study conducted on Pakistani population by Rafique et al.^10^ Correspondingly, in a in a Saudi Arabian cohort, Alzahrani et al observed a weak correlation between the agenesis of these tendons.^13^ Conversely, some studies have stated a significant association between PL and FDS absence. For instance, a study conducted on an Indian population found a statistically significant correlation between the absence of PL and FDS on the right side.^24^ These divergences may be credited to ethnic variations among ethnicities, size of sample and methodological differences across different populations.

### Strength of the study:

The present study contributes worthwhile information to the existing medical literature by providing comprehensive data on the prevalence and patterns of PL and FDS agenesis in a specific population. It accentuates the importance of taken into account geographical and ethnic factors in anatomical studies and emphasizes the need for clinicians to be mindful of such variations during physical examinations and surgical planning.

### Limitations:

The study was conducted at a single-centre; therefore, the results of the study cannot be generalized. In future, it is imperative to conduct similar research with a larger sample size at multiple centres. Additionally, comparative analysis can be planned with the help of ultrasonography to confirm clinical testing results.

## CONCLUSION

FDS functionality showed variability on two sides in terms of dependent and independent function. Both genders showed higher prevalence of right sided agenesis. Females showed more unilateral agenesis while male had higher bilateral prevalence. We found no association between agenesis of FDS of little finger with handedness and PL agenesis.

### Recommendations:

Including detailed anatomical knowledge of PL and FDS tendons into medical education can supplement surgical outcomes and patient care. During physical examinations, clinicians should assess the presence of PL and FDS tendons, especially when planning procedures that involve these structures. The knowledge of such absence is important for anatomists, ortho-surgeons, sports physicians, neuro and plastic-surgeons. Both PL and FDS absence have no effect on grip and pinch strength, the two tendons can be used for graft procedures and other associated corrective procedures. Likewise, the muscles tendon can also be used interchangeably in operative procedures when one of them is congenitally absent.^25^-^27^

### Author’s Contribution:

**QJ:** Conceived and designed the study, performed statistical analyses, drafted and edited the manuscript; responsible for the integrity of the research and approval of the final manuscript.

**LU:** Drafted the manuscript; responsible for the integrity of the research and final approval of the manuscript.
